# Occupational Therapy Students' Perceptions of the Role of Robots in the Care for Older People Living in the Community

**DOI:** 10.1155/2017/9592405

**Published:** 2017-02-07

**Authors:** Sławomir Tobis, Mirosława Cylkowska-Nowak, Katarzyna Wieczorowska-Tobis, Mariola Pawlaczyk, Aleksandra Suwalska

**Affiliations:** ^1^Laboratory of Occupational Therapy, Department of Geriatric Medicine and Gerontology, University of Medical Sciences, Poznan, Poland; ^2^Laboratory of Geriatrics, Department of Palliative Care, University of Medical Sciences, Poznan, Poland; ^3^Department of Geriatric Medicine and Gerontology, University of Medical Sciences, Poznan, Poland; ^4^Laboratory of Neuropsychobiology, Department of Psychiatry, University of Medical Sciences, Poznan, Poland

## Abstract

**Background:**

The question arises how recent developments in robotics can contribute to the care for older people. The study is part of the EU-funded ENRICHME project.

**Objectives of Study:**

The aim of the study was to investigate opinions of occupational therapy students (OTS), as future professional caregivers, on the use of robots in care for older people.

**Methods:**

It included 26 OTS from Poznan University of Medical Sciences. To collect data, the Users' Needs, Requirements, and Abilities Questionnaire (UNRAQ) was developed.

**Findings:**

OTS perceived the robot as “a useful device” and “an assistant” rather than “a companion” (*p* < 0.01). In their opinion, the most important functions of the robot were related to health aspects (emergency alarms, health parameters monitoring, physical activity and memory training, and reminders about medication, drinks, etc.), scored positively by 23–26 OTS. Functions such as mood detection, encouraging to contact with friends, and monitoring of food consumption were accepted by 16-17 OTS. Two statements concerning social functions (accompanying in everyday activities and decreasing the sense of loneliness) were rated positively by less the than half of the participants.

**Limitations and Recommendations for Further Research:**

A module concerning technology use, including robotics, should constitute an important part of the curricula of both academic and continuous education of OTS.

## 1. Introduction

World's societies have been ageing rapidly over the last years and older people have already outnumbered the younger cohort. A shortage in both formal and informal caregivers as well as declining resources and capacities is commonly observed [1]. Increasing number of individuals in need of care and limited availability of care professionals call for an implementation of new solutions, allowing for a reduction on the burden of caregivers who are active within the system. Such solutions could be based on the use of robots which would be involved in care and support for older persons living in the community.

If the systems including robots, addressed to the elderly, are to be effective, they have to meet the end users' unique needs and expectations: the health and social ones along with those related to the functioning of the individual in a particular cultural context [2]. For this purpose, the role of the robot must be defined comprehensively, also taking complex ethical and legal issues into consideration.

Similar to health information technology, it is crucial to understand a priori what factors are important in predicting older people's willingness to use robots' assistance [3]. Therefore, caregivers should be aware of those factors as they help promote the use of robots with community-dwelling older adults. Smarr et al. [4] found that older Americans were very imaginative regarding what the robot could do to assist them and were engaged, active, and motivated to discuss the robot's issues. In that study, they even preferred robot's assistance over human assistance, not only in many instrumental activities of daily living (e.g., medication reminder) but also in more complex activities such as new learning and hobbies.

While the majority of available papers are dedicated to the attitude of older people or their potential informal caregivers towards the robot, analysing particularly their expectations and determinants of acceptance [5–7], literature concerning the staff's point of view is sparse. This is surprising, as it is the formal caregivers who are essential to successful implementation and usage of a robot in the older persons' living environments, that is the application of “ageing in place.” This term acknowledges that the majority of older people are living independently in their own homes and want to stay there as long as possible, in familiar surroundings, with friends and family [8].

One of the few papers concerning professional caregivers in this aspect was published by Saito et al. who showed that nurses' stress levels at work were lower after the introduction of a seal-shaped “mental commit” robot PARO [9]. In focus groups studies by Zsiga et al. [10], professional caregivers showed a curious and open attitude towards the use of a robot.

In the field of occupational therapy, robotics in the literature is commonly attributed to rehabilitation tasks, mainly within the upper limb function [11, 12]. Many therapeutic techniques consist of showing movements to patients, guiding them through the movement, providing feedback on adequacy of performance, and, then, encouraging the patients to repeat the task until it has been accomplished. Moreover, the occupational therapist takes care of the cognitive status of the subject and provides appropriate exercises for their state [13]. As the key role of occupational therapists in patient-directed application of “ageing in place” is straightforward [14, 15], their interaction with the robot, focused on reaching therapeutic goals in care for older people, is of great importance and should be asked about. This observation was the starting point for our study.

## 2. Method

The project was approved by the Bioethical Committee of Poznan University of Medical Sciences, Poland (number 785/16).

The study is part of the ENRICHME project (*ENabling Robot and Assisted Living Environment for Independent Care and Health Monitoring of the Elderly*, financed by the European Union within the Horizon 2020 program, 643691) which tackles the progressive decline of cognitive capacity in the ageing population, adopting an integrated platform with a service robot for long-term human monitoring and interaction.

### 2.1. Participants

The study group consisted of occupational therapy students from Poznan University of Medical Sciences, as potential future formal caregivers for older people. We included only those who were close to finishing their 3-year bachelor curriculum and thus had substantial knowledge of their field. Twenty-six students out of 36 (more than 70%) participated in the study.

### 2.2. Procedure

To collect the data related to requirements for the robot, the Users' Needs, Requirements, and Abilities Questionnaire (UNRAQ) was developed, based on literature review and the expertise of the ENRICHME project partners. The UNRAQ is a structured questionnaire composed of fixed items used to gather data in the quantitative methodology, which enables categorisation of the answers and their analysis using statistical methods [16]. After a discussion of ENRICHME experts, it was decided to also add an opportunity for participants to comment on each item, as every single contribution of a participant may be of importance and of value while designing the robot. Subsequently, the creativity box was added at the end of UNRAQ for this aim. Therewith, we added elements of qualitative methodology to the questionnaire.

The UNRAQ is composed of three parts. The introductory part provides demographic information of the respondents (e.g., their age, education, and profession). The next section of the questionnaire is constructed of statements to which the participant is asked to express his/her level of agreement-disagreement. These statements pertain to four areas: the interaction with the robot, the role of the robot, social aspects, and assistive role. Answers are structured using 5-point Likert scale (I strongly disagree, I partially disagree, I neither agree nor disagree, I partially agree, and I strongly agree, scored in the range 1–5). This allows for the presentation of results in the form of a mean and a standard deviation (SD). Scores 1-2 are considered negative, score 3 is considered neutral, and scores 4-5 are considered positive. Given the number of participants (26), we used the following categorisation of their agreement, based on the results obtained: excellent agreement (all or all but one agreed, 26 or 25), very good agreement (24 or 23 agreed), and good agreement (17 or 16 agreed).

The final part of UNRAQ is the creativity box. Its purpose is to create an opportunity for the participants to speak out freely. The participant writes down in the creativity box all his/her ideas/suggestions for other functions the robot might have. They are asked to report any observations that come to mind while answering the questionnaire and are not already mentioned as comments to the statements. The free text remarks cited in the following parts of the paper come from both sources (comments to statements and creativity box).

Before completing the questionnaire, pictures of the Kompaï robot [17] were shown to provide the students with a model of the robot concept.

### 2.3. Statistical Analysis

The statistical analysis was performed with STATISTICA 12.0 software (StatSoft, Poland). Variables were expressed as percentages, frequencies, means ± standard deviation (SD), and medians. Normality in the data distribution was examined with Shapiro-Wilk's test.

Comparison between groups of paired data was made with the Wilcoxon test and differences in the distribution of quality variables between groups, with the *χ*^2^ test with Yates correction due to small sample size. *p* < 0.05 was considered statistically significant.

## 3. Results

The mean age of studied group was 21.6 ± 1.2 years (median: 22 years). All students stated that they were familiar with computers and all but one declared being familiar with working with technological systems. Four of them were active as informal caregivers for members of their families.

### 3.1. Preparedness for the Robot

As for the assessment of the preparedness of older persons for coping with the robot, the majority of students viewed them as unprepared (22 scored the statement* the elderly are prepared to interact with the robot* negatively: 10 strongly disagreed and 12 partially disagreed). The mean score for this statement was only 2.0 ± 1.1 (median 2.0). Nonetheless, the participants were of the opinion that this can be changed, “training on operation of the robot is necessary,” and that the introduction of a robot in the life of an older person should be “gradual, allowing for gaining familiarity, increasing the number of available functions.” In addition, the students stated that the robot should be “customised and programmed for every individual, since everybody has different needs.”

### 3.2. The Role of the Robot

**Figure 1:**
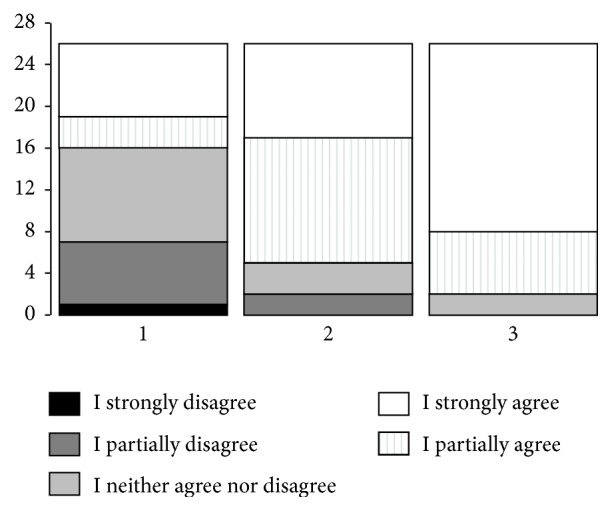
Detailed opinions of the participants related to following statements: (1) The robot should be a companion of the elderly person. (2) The robot should be an assistant of the elderly person. (3) The robot should be a useful device of the elderly person (something to be used when needed, with no other interaction).

The statement* the robot should be a companion of the elderly person* was rated positively by 10 students only. On the other hand, the statements* the robot should be a useful device of the elderly person (something to be used when needed, with no other interaction)* and* the robot should be an assistant of the elderly person* were rated positively more often (24 out of 26 and 21 out of 26 individuals strongly agreed with the above statements, *p* < 0.0001 and *p* = 0.0042, resp.). Detailed characteristics are presented in Figure 1.

All analysed functionalities of the robot were viewed favourable with the participants (which was expressed by mean scores above 3.0, Table 1). Seven statements gained positive scores from 25-26 participants (excellent agreement), another seven had 23-24 positive scores (very good agreement), further four were scored positively by 16-17 students (good agreement), and the remaining two were scored positively by 12 students.

Statements with good agreement were related to such functions as mood detection or encouraging to contact with friends and monitoring of food consumption.

In the comments, it was stressed that “the robot would be a potent helper; thank that the caregivers could have more time for the caretakers.” On the other hand, it was noted that “the robot should not replace the older person in all activities, as it might lead to higher dependency;” an attitude like “why perform an activity if the robot can do it” could lead to older adults taking a less active role in day to day care and having a potential negative impact on their overall health.

There were as little as two statements which were rated positively by less than half of the students:* the robot should accompany the owner in everyday activities (watching TV, preparing meals)* and* the robot could decrease the sense of loneliness and improve the mood of the elderly person* (Table 1). Notably, as many as nine students had neutral opinion about both these statements. The participants stated “the robot will not replace [human companionship]” but also “it should not be with the older person 24/7 as it would rather be a kind of supervision than company.” As far as the reminding function of the robot is concerned, the reminding about fluids intake gained special attention.

The participants broadly accepted the idea of the robot monitoring the health status of its elderly user and collecting their medical history (“the robot should be the source of information – e.g., in case of an emergency team call it could accelerate the help by immediately supplying parameters stored in its memory”). The students also expressed that the robot should react to certain conditions and trigger an alert accordingly (“my grandmother fell, stood up and stated that everything was fine, no need for emergency team, yet afterwards it showed she had a broken leg; the robot should alert that something happened even if seemingly all is OK”).

## 4. Discussion

Occupational therapists facilitate independence and support participation in occupations that are personally meaningful to clients, to enhance the wellbeing and quality of life. One of the core values supporting occupational therapy practice is viewing the patient as a person who acts on the environment [18]. With more and more technology becoming part of a human environment, it is imperative to think of integrating modern technology in the way occupational therapy helps its clients. The progress made in robotics in recent years makes it imaginable to involve robots in various fields of occupational therapy. We thus asked students approaching the end of their occupational therapy curriculum, as the ones who are likely to be soon confronted with this technology in their professional career, about their opinions on the use of robots in elderly care. So far, as suggested by Yerxa [18], the therapists perceived practice as “the profession.”

Peek et al. [19], based on literature review, stated that preimplementation acceptance of innovative solutions depends on both the perception of technology (concerns and benefits) and the necessity of technology (need and available alternatives). According to Broadbent et al. [8], healthcare robots can be broadly categorised into those that provide physical assistance, those that provide companionship, and those that monitor health and safety; however, some robotic systems cover more than one of these categories. The students mainly perceived the robot as “a useful device” and “an assistant,” significantly more often than “a companion.” Additionally, the only statements that were rated positively by less than half of the participants were* the robot could decrease the sense of loneliness and improve the mood of an elderly person* and* the robot should accompany the owner in everyday activities (like watching TV, preparing meals)*. The participants did not seem to express the expectations that the technology at its current level would be sufficient to allow the robot to engage socially in a successful manner. This may, however, also indicate the students' focus on the tasks rather than the social aspects of technology use and may result from their young age/perspective of life. Subsequently, the assistive and monitoring functionalities of the robot were rated higher than the social ones. A comparable pattern of distribution of interests among the young was observed by Zsiga et al. [10].

One may thus speculate that students perceive the robot as a therapy aide, not a substitute for a caregiver, which was additionally reflected in general remarks like “the robot will never replace a human.” Similar attitudes were observed by Boissy et al. [20] in focus groups of professional caregivers (among whom there was one occupational therapist) and older persons: “a robotic telepresence service would not replace healthcare professionals or family members, but could supplement them in providing care.” In our study, the students noticed that the use of a robot in repeatable activities would allow them to devote more time to their clients. They thus do not have (or at least do not express) the fear of being professionally replaced by the robot. The lack of a feeling of one's job being threatened finds reflection in the study of Frey and Osborne [21], according to which occupational therapy is the profession sixth least endangered by the introduction of computers and robots, out of 702 rated. Dijkers et al. [22] also noted that occupational therapists can quickly adjust to working effectively with this type of equipment.

Wu et al. [23] defined common barriers to robot's acceptance which are uneasiness with technology, feeling of stigmatisation, and ethical issues such as “use or lose it logic” (e.g., if a robot does things for its user, does the user lose some capacities because of not making the effort?). In our study, we found indications of all of them. The students pointed to the necessity of competent pretraining for the older persons for coping with the robot, as they would lack the necessary knowledge and/or experience with technology. They also signalled the risk of losing abilities when certain tasks would be performed by the robot instead of the older subject.

Among the highest scored functions of the robot (25 positive out of 26) were reminders about appointments, medication, and meal times, which fits into the holistic approach to the client, as it encompasses the most significant issues of older individuals. Among them, the danger of dehydration was specifically pointed out as needing special attention. In our opinion, this is worth underlining because dehydration is a common (and often underestimated) risk factor of increasing dependence in age.

Our study has some limitations. The studied population is small, yet we managed to include over 70% of the whole students group, which is a fairly good response rate. In addition, the Poznan University of Medical Sciences is the only university level medical school throughout the country offering a curriculum in occupational therapy. Our students are uniquely prepared to work as members of interprofessional geriatric teams. Secondly, our study was theoretical in the sense that the participants did not have prior interactions with a healthcare robot. It was part of the ENRICHME project, aimed at the definition of necessary and desirable functionalities of the robot to be implemented in the community. Thus, in the next iteration, the participants will have the opportunity to interact with the robot in a real world context which will constitute the base for further investigation of the topic.

Occupational therapists must understand the patient's challenges and/or limitations, have knowledge of different treatment modalities and approaches, and be able to apply them creatively [22]. As robotics makes more therapeutic applications available, both occupational therapy curriculum and continuous education opportunities should take into account the availability of increasingly intelligent robots and how they can help occupational therapy best meet their patients' needs.

## Figures and Tables

**Table 1:** Quantitative analysis of the opinions of occupational therapy students related to the statements about the role of the robot in the care for older people, arranged by *number of students who agreed with the statement* and *mean score*.

Statement	Number of students who agreed with the statement (strongly or partially)	The score (mean ± SD; median)
*The robot should call the centre in case of emergency*	26	4.8 ± 0.4; 5
*The robot should increase the safety of the elderly: calling for help when needed, monitoring health parameters (blood pressure, heart rate, etc.)*	26	4.8 ± 0.4; 5
*The robot should encourage and guide the elderly to perform physical exercises*	26	4.6 ± 0.5; 5
*The robot should remind the elderly about medication*	25	4.8 ± 0.5; 5
*The robot should remind the elderly about appointments*	25	4.5 ± 0.6; 5
*The robot should have entertainment functions (e.g. gaming partner, reading aloud or playing music function)*	25	4.4 ± 0.6; 4
*The robot should remind about meals times, drinks*	25	4.4 ± 0.6; 4
*The robot should help the elderly to preserve their memory function for example by playing memory games with them*	24	4.6 ± 0.6; 5
*The robot should measure physiological parameters (blood pressure, heart rate, body temperature) of the elderly person*	24	4.5 ± 0.6; 5
*The robot should observe the behaviour of the elderly person to detect falls or changes due to illness*	24	4.5 ± 0.6; 5
*The robot should monitor the environment (temperature, humidity) and suggest air conditioning adjustment or windows opening*	24	4.5 ± 0.6; 5
*The robot should increase the safety of the elderly home: for example locking doors, detecting leaking gas and so forth*	23	4.5 ± 0.8; 5
*The robot should help the owner to find lost objects (e.g. glasses, keys)*	23	4.4 ± 0.7; 5
*The robot should provide advice about a healthy diet*	23	4.3 ± 0.7; 4
*The robot should monitor the amount of food and fluid intake of the owner*	17	3.8 ± 1.1; 4
*The robot could encourage the elderly to enhance their contacts with friends*	17	3.9 ± 1.0; 4
*The robot should detect the owner's mood (facial expression)*	17	4.0 ± 1.1; 4
*The robot should initiate contacts with others (calling friends, initiating skype conversations)*	16	3.7 ± 1.4; 4
*The robot should accompany the owner in everyday activities (watching TV, preparing meals)*	12	3.6 ± 1.2; 3
*The robot could decrease the sense of loneliness and improve the mood of the elderly person*	12	3.4 ± 1.2; 3
